# Association between the awareness of osteoporosis and the quality of care for bone health among Korean women with osteoporosis

**DOI:** 10.1186/1471-2474-15-334

**Published:** 2014-10-04

**Authors:** Hyun-Young Shin, Hee Cheol Kang, Kiheon Lee, Sang Min Park

**Affiliations:** Department of Family Medicine, Severance Hospital, Yonsei University College of Medicine, Seoul, South Korea; Department of Family Medicine, Seoul National University Bundang Hospital, Seoul National University College of Medicine, Gyeonggi-Do, South Korea; Department of Family Medicine, Seoul National University Hospital, Seoul National University College of Medicine, Seoul, South Korea

**Keywords:** Awareness, Bone health behavior, Bone health, Bone health quality, Osteoporosis, Treatment of osteoporosis

## Abstract

**Background:**

The prevalence of osteoporosis is increasing and is a socio-economic burden worldwide. Although screening tests for osteoporosis in Korea are easily accessible, this condition remains undertreated. Evaluating post-diagnostic behavior changes may be helpful for improving the quality of care for bone health in osteoporotic patients.

**Methods:**

After reviewing the Fourth Korean National Health and Nutrition Examination Survey 2008–2009, 1,114 women with osteoporosis aged >50 years were included in this cross-sectional study. Factors related to bone health were categorized into the following groups: (1) behavioral health (smoking, alcohol consumption, and physical activity); (2) measured factors (lean body mass [kg], appendicular skeletal muscle mass [kg], and serum vitamin D level [nmol/L]); and (3) nutritional factors (calcium intake, vitamin/mineral supplementation, and healthy supplementary food). Logistic regression analysis and analysis of covariance was conducted after adjusting for age, education, income, residential area, height, weight, and self-perceived health using a weighted method.

**Results:**

Doctors diagnosed 39.5% of patients with osteoporosis, and these patients were compared with the control group. The awareness group, who had been diagnosed with osteoporosis by a doctor, had a lower proportion of smokers and higher serum vitamin D level than the control group, who had never been diagnosed with osteoporosis. No other associations were found for quality of bone health care variables. The awareness group had higher odds ratios of vitamin/mineral replacement and healthy supplementary food but no other differences were observed, indicating the patients’ beliefs in bone health care do not follow the recommended clinical guidelines (e.g. higher physical activity, lower alcohol consumption).

**Conclusion:**

To improve the quality of care for bone health in osteoporotic patients, an initial step should be the development of post-diagnostic procedures such as patient counseling and education through a multi-team care approach.

**Electronic supplementary material:**

The online version of this article (doi:10.1186/1471-2474-15-334) contains supplementary material, which is available to authorized users.

## Background

Osteoporosis is a common chronic disease in aging populations with an increasing incidence worldwide[1]. Since baby boomers in Korea are aging, issues related to bone health and chronic conditions have not only become medical problems, but also a socio-economic burden[2]. Despite the increase in its significance, osteoporosis is widely recognized as a preventable and treatable disease; therefore, an appropriate detection and management system that includes lifestyle modifications may minimize the burden on public health resources worldwide. Although clinical guidelines recommend cessation of tobacco use, avoidance of excessive alcohol intake, participation in regular exercise, and an adequate intake of calcium and vitamin D for maintaining bone health[3], several studies have shown that osteoporosis patients do not follow the recommended clinical guidelines after diagnosis of the disease[4].

In Korea, the national health care system enables people to easily access medical institutions where they can undergo dual energy x-ray absorptiometry (DEXA) scans to screen for low bone mineral density (BMD), which can indicate osteoporosis[5]. However, there is a lack of standardized post-diagnostic and follow-up care, which consequently results in the undertreatment of osteoporosis[6, 7]. Moreover, there is a lack of studies reporting on the compliance of clinical guidelines for bone health behavior following a diagnosis of osteoporosis in Korea. Understanding which bone health behaviors are well performed or not may be helpful in the implementation of education or intervention programs to improve the bone health status of osteoporosis patients. Therefore, we analyzed the quality of care for bone health in relation to patients’ awareness of their disease using data from the Fourth Korean National Health and Nutrition Examination Survey 2008–2009 (KNHANES IV).

## Methods

### Study population

KNHANES IV (2008–2009)[8] is a nationwide survey representing the general Korean population, and includes comprehensive information on the health status, health behavior, and socio-demographics of 21,517 individuals in 576 national districts. A stratified multistage probability sampling design was used. The health interview survey of KNHANES IV was conducted through face-to-face interviews at patients’ homes by trained interviewers. Each patient provided informed consent prior to inclusion in the study. Initial candidates for this study were 11,064 women who completed the health interview and health examination surveys. Individuals aged ≥50 years who underwent a DEXA scan and completed the assessment of awareness of osteoporosis were then selected; 1,114 women were included in the final study population.^7^ This study has adhered to the STROBE guidelines and was approved by the Institutional Review Board of the Korea Centers for Disease Control and Prevention.

### Bone mineral density measurement

The bone mineral density (BMD; g/cm^2^) measurements of patients’ lumbar spine and femoral neck were obtained using a DEXA scanner (DISCOVERY-W fan-beam densitometer, Hologic Inc., Bedford, MA, USA). The coefficient of variation (CV) of the BMD measurement based on reproducibility scans was 1.9% for the L1–4 spine and 2.5% for the femoral neck. We used the L1–4 and femoral neck values for BMD analysis.

The definition of osteoporosis was based on the World Health Organization’s T-score criteria (T-score ≤ −2.5)[9], and we used the maximum BMD value for Japanese patients as a reference owing to the lack of established Korean diagnostic criteria[10]. If a patient had a low T-score from one of the BMD measurements of the lumbar spine or femoral neck (or both), they were classified as having osteoporosis. A medical history of fractures was not used to define osteoporosis, as KNHANES IV included neither confirmatory imaging tests nor a means of distinguishing between low- and high-energy fractures. In addition, we considered patients who answered “yes” to the question “are you taking prescription medication for osteoporosis?” as having osteoporosis because of the possibility that the medication had increased their BMD. As part of KNHANES IV, osteoporotic patients with BMD T-scores ≤ −2.5 or with radiography-confirmed fractures were eligible to receive reimbursement for the cost of anti-osteoporotic drugs (e.g., bisphosphonate, raloxifene, or hormonal agents) under National Health Insurance coverage in Korea.

### Definition of osteoporosis awareness and treatment

Osteoporosis awareness was assessed using the question, “have you been diagnosed with osteoporosis?” Patients who answered yes were considered aware they had osteoporosis and were included in the awareness group. Patients diagnosed with osteoporosis by DEXA results who answered no were included in the control group (unawareness group). Treatment of osteoporosis was defined as the self-reported use of a prescription medication for the management of osteoporosis at the time of the survey. In Korea, Food and Drug Administration (FDA)-approved bone-specific drugs, including bisphosphonate, raloxifene, hormones, parathyroid hormone, and calcitonin, require doctors’ prescriptions and are covered by National Health Insurance in the case of DEXA-confirmed osteoporosis. The reimbursement criteria for osteoporosis medication in Korea do not include other risk factors besides the T-score and history of fracture. In contrast, calcium and vitamin D supplementation are over-the-counter drugs and do not require prescriptions.

### Quality care indicators of bone health

From the KNHANES IV database, we collected information on various factors related to quality of care indicators for bone health, which has comprehensive meanings for improving bone health, including not only behaviors but also other measureable factors, and these were divided into three groups: health behaviors, measured factors, and nutritional factors.

### Health behaviors

Information on non-smoking or smoking status, regardless of the amount, was collected by a self-reporting questionnaire. Cigarette smoking causes a lower BMD due to an increased bone loss rate, and a higher fracture risk due to low BMD and poor neuromuscular function[11–13].

Not excessive alcohol consumption (<3 standard drinks [StDs] per occasion) and excessive alcohol consumption (≥3 StDs per occasion) data were also collected by a self-reporting questionnaire. Heavy alcohol consumption is associated with low bone mass due to direct toxic effects on the bone, as alcohol disrupts calcium and bone homeostasis, which facilitates the disturbance of bone growth and induces bone complications such as fractures[13–17].

Adequate physical activity (≥3,000 metabolic equivalent [MET]-min/week) or inadequate physical activity (<3,000 MET-min/week) data were collected. Exercise improves bone density and the rate of bone loss, which ultimately reduces falls and prevents fractures, thus aerobic, weight bearing (walking, stair climbing, jogging, dancing, and tennis), and resistance exercises (weight training and other resistive exercise) are recommended[18, 19].

### Measured factors

Lean body mass (kg) was calculated on the basis of data from DEXA scans by subtracting body fat and bone weight from total body weight and appendicular skeletal muscle mass to determine the sum of the muscle weight of the extremities (kg). Studies have reported a positive association between BMD and lean body mass, which may be due to mechanical load pressure on the skeleton, dynamic mechanical load from muscle contraction, and other determinants such as genetic, dietary, and hormonal factors[20–22]. Serum vitamin D levels (nmol/L) were analyzed by radioimmunoassay using the 1470 WIZARD gamma-Counter (PerkinElmer, Finland) and the 25-hydroxyvitamin D 125I RIA Kit (DiaSorin, USA). Vitamin D is an essential nutrient that can be obtained by ultraviolet light exposure or oral intake, and it helps to reduce the risk of fractures and improve BMD and the effective action of bisphosphonate by promoting calcium absorption and bone mineralization[23, 24].

### Nutritional factors

Adequate calcium intake (≥700 mg/day) or inadequate calcium intake (<700 mg/day) was monitored by a 24-h food recall and analyzed using the CAN-Pro software 3.0 (Korean Nutrition Society, Seoul, Korea). Calcium is an essential mineral for maintaining healthy skeletal structures, and is involved in bone mineralization and remodeling through bone absorption and resorption. Diet and supplements are sources of calcium that can help reduce the rate of bone loss, increase BMD, and reduce the risk of bone fractures[25–27].

Vitamin/mineral supplementation was examined by a self-reporting questionnaire that asked, “Within the past year, have you ever taken any kind of vitamin or mineral for more than 2 weeks?”. In Korea, vitamin/mineral supplementation is considered to be the first rank of dietary supplements[28], and among them, multi-vitamins and vitamin C are most commonly used[29].

Consumption of healthy supplementary foods was determined by responses to the question, “Within the past year, have you ever taken any kind of healthy supplementary foods for more than 2 weeks?”. In Korea, healthy supplementary foods can be circulated after receiving permission from the Korean FDA, and ginseng, followed by glucosamine and probiotics, are the most commonly used[30].

### Statistical analysis

We calculated the means with standard deviation, or the number of cases with the proportions of individuals aware of their condition according to the variable using a weighted population sample to reflect the sampling method and response rate. Student t-tests and chi-squared tests were used to compare mean values and percentages respectively, by osteoporosis awareness in Table 1. We used logistic regression analysis to determine which variables relating to quality of bone health care were associated with osteoporosis awareness, and each variable was adjusted for age only (Model 1). Next, we performed multivariate logistic regression analysis and adjusted for demographic factors including age, educational level (high school or higher, middle school, and elementary school or less), household monthly income status (upper, upper middle, lower middle, and lower), and residential area (urban or rural; Model 2). Then height, weight, and self-perceived health status were added to the adjusted variables (Model 3). Self-perceived health status was classified by 4 levels (very poor, poor, fair, or good) according to response to the question “How do you assess your own health status?”. Adjusted odds ratio [OR], 95% confidence intervals [CI], and p-values were calculated to show the strength of each association. Analysis of covariance was used to calculate mean levels of measured factors after adjusting for age, education, household income, residential area, height, weight, and self-perceived health status. A p-value <0.05 was considered significant. All statistical analyses were performed using SAS statistical software, version 9.2 (SAS Institute Inc., Cary, NC, USA).

**Table 1:** **Characterization of osteoporotic patients according to their awareness**

Total (n)	Awareness of osteoporosis^a^		
	No	Yes	
n = 1114	n = 646	n =468	p-value
n = 2.68^b^ (100)	n = 1.62^b^ (60.5)	n = 1.06^b^ (39.5)	
**Age** (year)	70.6 ± 0.53	67.8 ± 0.51	<0.01
**BMI** (kg/m^2^)	23.2 ± 0.14	23.7 ± 0.16	0.02
**Education**			0.39
Elementary school or lower	553 (82)	388 (77.7)	
Middle school	42 (7.6)	34 (9.2)	
High school or higher	45 (10.4)	46 (13.1)	
**Household Income**			0.05
Low	327 (45.8)	232 (44.4)	
Low middle	139 (21.8)	125 (28.0)	
Upper middle	97 (20.1)	53 (14.2)	
Upper	64 (12.2)	52 (13.4)	
**Residential area**			0.78
Rural	361 (69.5)	266 (68.6)	
Urban	285 (30.5)	202 (31.4)	
**Self-perceived Health Status**			< 0.01
Excellent/good	203 (35.4)	105 (23.7)	
Fair	148 (24.7)	106 (23.7)	
Poor/very poor	289 (39.8)	257 (52.7)	

n: unweighted sample size, ^a^For the question, “have you been diagnosed with osteoporosis?”, patients who answered yes were considered aware that they had osteoporosis and were included in the awareness group. Patients diagnosed with osteoporosis who answered no were included in the control group (unawareness group). n^b^: weighted sample size in millions.

BMI, body mass index.

## Results

In the present study, the changes in bone health behavior such as excessive alcohol consumption, adequate physical activity, body muscle mass, and adequate calcium intake were not significant among osteoporotic patients as we had hypothesized. On the contrary, vitamin/mineral replacement and healthy supplementary food intake were found to be significantly different for osteoporotic patients. Our findings, based on model 3 in Table 2 and3, are described below.

**Table 2:** **Behavioral risk factors related to bone health according to awareness of osteoporosis**

Awareness of osteoporosis^a^	No	Yes
Current smoker*		
No. of events (%)^b^	7.67	2.39
Model 1^§^	1.00	0.33 (0.17–0.67)
Model 2^**∥**^	1.00	0.28 (0.14–0.58)
Model 3^¶^	1.00	0.30 (0.14–0.61)
**Excessive alcohol consumption** ^**†**^		
No. of events (%)^b^	9.31	9.02
Model 1^§^	1.00	0.82 (0.46–1.45)
Model 2^**∥**^	1.00	0.87 (0.48–1.56)
Model 3^¶^	1.00	0.86 (0.47–1.58)
**Adequate physical activity** ^**‡**^		
No. of events (%)^b^	18.5	22.1
Model 1^§^	1.00	1.07 (0.75–1.53)
Model 2^**∥**^	1.00	1.03 (0.72–1.46)
Model 3^¶^	1.00	1.11 (0.77–1.59)

^a^In the question, “have you been diagnosed with osteoporosis?”, patients who answered yes were considered aware that they had osteoporosis and were included in the awareness group. Patients diagnosed with osteoporosis who answered no were included in the control group (unawareness group). ^b^Percentage of weighted sample size in millions; *Current Smoker: smoking currently; ^†^Excessive Alcohol Drinking: ≥3 standard drinks per occasion; ^‡^Adequate Physical Activity: ≥3,000 metabolic equivalent [MET]–min/week.

Adjusted Variable: ^§^Age; ^∥^Model 1 + education, household income, and residential area; ^¶^Model 2 + height, weight, and self-perceived health status.

**Table 3:** **Nutritional factors related to bone health according to awareness of osteoporosis**

Awareness of osteoporosis^a^	No	Yes
Adequate calcium intake*		
No. of events (%)^b^	8.47	10.2
Model 1^§^	1.00	1.15 (0.71–1.89)
Model 2^**∥**^	1.00	1.22 (0.74–2.00)
Model 3^¶^	1.00	1.19 (0.73–1.94)
**Vitamin/Mineral replacement** ^**†**^		
No. of events (%)^b^	18.5	25.9
Model 1^§^	1.00	1.50 (1.08–2.07)
Model 2^**∥**^	1.00	1.58 (1.15–2.18)
Model 3^¶^	1.00	1.60 (1.15–2.23)
**Healthy supplementary food** ^**‡**^		
No. of events (%)^b^	21.6	30.3
Model 1^§^	1.00	1.45 (1.00–2.11)
Model 2^**∥**^	1.00	1.46 (1.00–2.14)
Model 3^¶^	1.00	1.55 (1.06–2.26)

^a^For the question, “have you been diagnosed with osteoporosis?”, patients who answered yes were considered aware that they had osteoporosis and were included in the awareness group. Patients diagnosed with osteoporosis who answered no were included in the control group (unawareness group). ^b^Percentage of weighted sample size in millions; *Adequate calcium intake: ≥700 mg/day calcium intake; ^†^Vitamin/Mineral replacement: Yes or No; ^‡^Healthy Supplementary Food: Yes or No.

Adjusted variables: ^§^Age; ^∥^Model 1 + education, household income, residential area; ^¶^Model 2 + height, weight, self-perceived health status.

### Baseline characteristics of the study population

The characteristics of study patients are shown in Table 1. Among the 1,114 osteoporotic patients (2,680,000 people when weighted), 39.5% of patients were diagnosed with osteoporosis by doctors. The mean age of the awareness group was lower than that of the control group (67.8 vs. 70.6 years respectively; p < 0.01), and the mean BMI of the awareness group was higher than that of the control group (23.7 vs. 23.2 kg/m^2^ respectively; p = 0.02). As for self-perceived health status, 52.7% of the awareness group responded that their status was poor or very poor, while 39.8% of the control group provided the same response (p < 0.01).

### Behavioral risk factors for bone health

Logistic regressions presented in Table 2 show that patients in the awareness group had a lower current smoking rate than those in the control group (OR 0.30, 95% CI: 0.14–0.61). However, no significant differences were found for alcohol consumption and physical activity between the awareness and control groups.

### Measured factors of bone health

Lean body mass and appendicular skeletal muscle mass were calculated to determine whether there was an association between osteoporosis awareness and muscle volume. Between the awareness and control groups, there were no significant differences in the adjusted means of lean body mass and appendicular skeletal muscle mass (31.2 ± 0.1 vs. 31.1 ± 0.1 kg, p = 0.57; 13.3 ± 0.1 vs. 13.3 ± 0.1 kg, p = 0.53 respectively). In contrast, serum vitamin D level in the awareness group was higher than that in the control group (49.7 vs. 44.9 nmol/L, p < 0.01).

### Nutritional factors and bone health

Although there was no significant difference for adequate calcium intake, the patients in the awareness group showed higher ORs for vitamin and mineral replacement (OR 1.60, CI: 1.15–2.23) and healthy supplementary food (OR 1.55, 95% CI: 1.06–2.26) than the control group, as shown in Table 3.

## Discussion

Our study revealed that there were only a small number of differences in bone health behaviors between the groups of Korean women aged >50 years who were aware of having osteoporosis and those who were unaware. We found that 39.5% of all osteoporotic patients in our sample were aware of their diagnosis by doctors. Surprisingly, our results showed that when considering most variables, the awareness group did not show better bone health promoting behavior than the unaware group. The only beneficial behavioral factor that was present among the awareness group but not the unawareness group was a lower rate of smoking, though this may not be caused by being aware of their osteoporotic condition. Otherwise, there were no significant differences in alcohol consumption and physical exercise between the two groups.

Muscle mass (lean body mass or appendicular skeletal muscle mass) is an important factor representing the amount of muscle-strengthening exercise conducted, and it is a protective factor against bone fractures. Several studies have reported a positive correlation between lean body mass and BMD, which suggests that the muscle exerts mechanical load forces on the bone and is therefore a metabolically active organ affecting the bone[31, 32]. In our study, there was no difference between the two groups for lean body mass and appendicular skeletal muscle mass after adjusting for multiple variables including weight. These results suggest that osteoporosis did not change their behavior by engaging in adequate physical activity, especially muscle-strengthening exercise. Therefore, this information should be stressed not only to patients and people at risk of osteoporosis, but also to healthcare providers.

Calcium and vitamin D are essential for reducing fracture risk and improving BMD and the effective action of bisphosphonate therapy[33, 34]. In our study, there was no difference between the awareness and control groups for adequate calcium intake (>700 mg/day). Mean serum vitamin D level was higher in the awareness group than the control group. However, both groups contained some patients with hypovitaminosis D (cut off level 74.9 nmol/L)[34]. This discrepant result might be due to the fact that calcium is supplied only through dietary intake, whereas vitamin D is acquired from both nutrition and cutaneous synthesis. Alternatively, the different measurement methods used (intake of calcium g/day compared to serum vitamin D levels) could be the reason for this result. Interestingly, the ORs for vitamin/mineral replacement and healthy supplementary food intake were significantly higher for the awareness group. This result indicates a distortion in bone health behavior, as well as a lack of proper osteoporosis education and instruction from healthcare providers. Correcting these inaccurate perceptions and providing adequate information on osteoporosis will be critical in guiding patients towards better bone health behaviors[35].

The National Bone Health Alliance in the U.S.[36] is a good example of the effort to establish awareness on osteoporosis management, and educational materials, programs, and effective campaigns need to be developed. A Korean study demonstrated that consultation with a doctor following a diagnosis of osteoporosis and osteopenia was the only factor associated with receiving treatment[37], which signifies the important role of healthcare providers. A multipronged systematic team approach and better cooperation between primary and secondary care providers would create fully integrated care for patients, which could lead to improved awareness of the under-recognition and undertreatment of osteoporosis. Additionally, post-diagnostic procedures, providing the correct insights regarding management guidelines for medical professionals, and a continuous monitoring and support system for patients are required. The utilization of tools for the assessment of osteoporotic risk, such as the scorecard system for osteoporosis in Europe[38], the Physician Quality Reporting Initiative[39], and the Korean Osteoporosis Risk-Assessment Model[40], as well as the application of these to the electronic medical record system could lead to increased awareness.

This study has several limitations. Firstly, it has a cross-sectional design, so it is difficult to determine the causality of any relationship. Secondly, the maximum BMD value for Japanese patients was used as a reference owing to the lack of established Korean diagnostic criteria. Thirdly, there may be reporting bias, as data were collected through self-reporting questionnaires, including data on osteoporotic medication. In spite of these limitations, to our knowledge, this is the first study to examine the features of the awareness group of osteoporotic patients based on national data from Korea.

## Conclusions

Our study of Korean female osteoporotic patients demonstrated that awareness of osteoporosis does not guarantee a patient will practice good bone health behavior, except in regular smokers, who ceased smoking. In addition, the awareness group, who answered yes to the question, “have you been diagnosed with osteoporosis?”, showed inappropriate behavior to enhance bone health (such as increasing physical activity and reducing excessive alcohol intake) and distorted nutritional behavior in relation to taking supplementary vitamins and healthy supplementary food. Developing post-diagnostic procedures that include patient counseling and education through a multi-team care approach should be an initial step in guiding patients towards high quality bone health. Furthermore, large prospective studies are needed to establish more reliable, strategic, and practical plans for osteoporotic patients in the future.

## Authors’ original submitted files for images

Below are the links to the authors’ original submitted files for images. Authors’ original file for figure 1 Authors’ original file for figure 2

## Abbreviations

BMD: Bone mineral density
CV: Coefficient of variation
FDA: Food and Drug Administration
MET: Metabolic equivalent
OR: Odds ratio
StD: Standard drink.
