# Supplementary material for: Association between the awareness of osteoporosis and the quality of care for bone health among Korean women with osteoporosis
Source: BMC Musculoskelet Disord. 2014 Oct 4;15:334. doi: 10.1186/1471-2474-15-334 (PMC4194369; doi:10.1186/1471-2474-15-334)
Supplement: Supplementary file 1 — Authors’ original file for figure 1 [file 12891_2014_2266_MOESM1_ESM.pdf]

Figure 1 Adjusted means for lean body and appendicular skeletal muscle mass in two osteoporotic groups

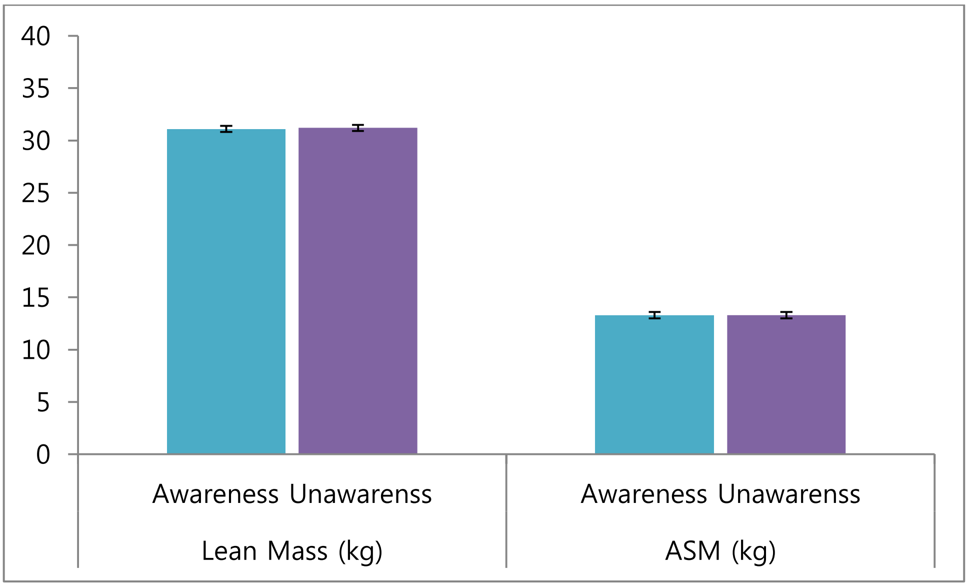

Adjusted variables: age, education, household income, residential area, height, weight, and self-perceived health status

ASM, appendicular skeletal muscle mass
